# Negative Experiences of Non-Drinking College Students in Great Britain: an Interpretative Phenomenological Analysis

**DOI:** 10.1007/s11469-017-9848-6

**Published:** 2017-12-04

**Authors:** Lisa Jacobs, Dominic Conroy, Adrian Parke

**Affiliations:** 1 Forensic and Clinical Research Group, School of Psychology, University of Lincoln, Sarah Swift Building, Brayford Wharf East, Lincoln, LN5 7AY UK; 2 School of Psychology, Birkbeck University London, London, UK

**Keywords:** Interpretative phenomenological analysis, Alcohol, Non-drinking, College students, Females

## Abstract

Research relating to alcohol use amongst university students primarily examines the effects of binge drinking. Researchers rarely focus on a range of drinking styles including light or non-drinking. This study was designed to gain an in-depth understanding of the lived experiences of female, first year UK undergraduates, who do not drink alcohol. Semi-structured interviews were conducted with eight participants. Narratives were analysed using interpretative phenomenological analysis (IPA; by Smith and Osborn (Sage 51-80, 2003). Three superordinate themes were identified: “managing the feeling that you don’t belong” highlights the importance of managing social interactions as a non-drinker; “experiencing social exclusion” recognises the impact on social bonding as a result of insufficient socialising opportunities; and “experiencing peer pressure and social stigma” highlights the scrutiny and labelling participants endured. These findings provide an understanding of some of the difficulties experienced by these undergraduates as a result of their non-drinking status. Implications of this research are discussed and areas for future research are outlined.

Excessive alcohol consumption is a major health concern in the UK, with drinking playing a significant social role in British culture (Holmes et al. 2016). In response, the UK government has recently lowered the recommended alcohol intake to 14 units of alcohol per week for both men and women (Drinkaware 2016). Heavy alcohol consumption and binge drinking amongst university students is common, with many students consuming alcohol in excess of recommended drinking guidelines (Craigs et al. 2011). It has been reported that 52% of heavy drinking students do not acknowledge the dangers of high alcohol consumption levels (Longstaff et al. 2014), and despite witnessing negative aspects of alcohol use, such as peer violence and health implications, these consequences are not severe enough to motivate behaviour change amongst young people (de Visser et al. 2013).

Research has highlighted the importance of alcohol as a medium for facilitating social activity amongst young people; it can help to overcome shyness, and aid the bonding process, thereby helping to promote a sense of belongingness (de Visser et al. 2013). Features of the university campus environment can compound heavy alcohol use being regarded as a normative and therefore acceptable behaviour amongst students. For example, drink-related activities in connection with “Fresher’s Week” are widely recognised amongst first year undergraduates as an important initial point of social contact with peers during the first week of the academic term (Coleman 2012). With peer acceptance fundamental to university life, many undergraduates are motivated to drink alcohol to enhance their ability to socialise with their peers (Davoren et al. 2016), helping to increase perceived self-confidence (AERC 2010) and reduce social inhibition (Penny and Armstrong-Hallam 2010). Furthermore, young people emphasise the value of being identified as a drinker; with non-drinking considered abnormal, individuals feel that by starting drinking, this will alleviate the risk of being labelled as boring (de Visser et al. 2013).

However, evidence suggests that the complexion of drinking patterns amongst young adults in the UK are changing. For example, between 2005 and 2013, individuals aged between 16 and 24 who reported abstinence from alcohol had risen by 40% (Office for National Statistics 2015). Of interest here is whether these societal changes are reflected in changes in how heavy alcohol consumption is viewed in terms of social acceptability amongst university students. Therein, more research on varied drinking styles (e.g., moderate drinking, non-drinking) may be timely, giving a sense of how different drinking approaches are experienced and viewed in university settings. One way in which such research can proceed is by exploring the challenges of being a non-drinking first year student in the UK, given that these individuals can be understood to be defying cultural norms and resisting peer pressure to drink.

Previous literature has explored non-drinking amongst both young adults generally (Herring et al. 2014) and undergraduate students across all university year groups (Conroy and de Visser 2014; Piacentini and Banister 2009); however, research has not yet focused specifically on the experiences of first year undergraduate students who have just started encountering the university drinking culture. First year undergraduates face considerable personal, social, and academic pressure during a unique life transition period as documented in various international studies of university student experiences (Borsari et al. 2007); therefore, it is pivotal to explore the experiences of students who also may also face the added challenge concerned with being a non-drinking first year student.

Several studies have emphasised that abstinence from alcohol for a young person may be difficult, given its social significance and relationship with feelings of social inclusion (Frederiksen et al. 2012). Conroy and de Visser (2014) also supported this view, highlighting that the absence of alcohol would negatively impact the ability to make friends. In addition to social implications, research suggests that non-drinking may hold implications for gender identity. Heavy alcohol consumption and public drinking have traditionally been associated with men, holding associations with masculinity, with being able to withstand large quantities of alcohol important for displaying masculine competency (de Visser and Smith 2007). However, in the last 30 years, women’s alcohol consumption levels have increased, with the gap between heavy drinking patterns between men and women narrowing. Women drinkers were traditionally stigmatised as lacking femininity, respectability, and neglectful of their caregiving role (Day et al. 2004), but currently, women’s drinking is deemed as more normalised, pleasurable, and a key component of female socialising and bonding (Lyons and Willot 2008). Despite becoming increasingly more tolerated, excessive drinking amongst women has also received media scrutiny. Research indicated that female drinking is perceived as an attempt to adopt masculine characteristics. Female drinking resulted in being labelled a “ladette”, defying traditional gender norms (Day et al. 2004).

There are still distinctions between whether non-drinking is an appropriate behaviour amongst men and women. Research highlighted that abstinence from alcohol for males may be seen as a failure to uphold a masculine identity, resulting in implications relating to inferiority or prompting questions regarding homosexuality (Conroy and de Visser 2013; de Visser et al. 2009). In comparison, research has indicated that it may potentially be more socially acceptable for women to be non-drinkers, rather than men, with female non-drinkers often praised or remaining inclusive within social groups (Conroy and de Visser 2013).

Previous literature that has explored the experiences of non-drinkers and managing anti-consumption have commonly recruited mixed gender samples and have explored the cultural associations between heavy drinking and masculinity (Conroy and de Visser 2013); however, literature has not yet focused solely on female non-drinkers. Paying particular attention to the experiences of non-drinking female undergraduates is warranted, given that female non-drinkers might undergo unique consequences to socialising sober. For example, research has indicated that women have an instinctual need to be the caretaker to those who are drinking too much and passing out (Howard et al. 2007). Furthermore, statistics from Drinkaware indicate that 54% of female students have experienced sexual harassment on a night out (The Telegraph 2016). Given that alcohol has been associated with a reduced ability to evaluate risk, including the ability to recognise ambiguous social cues that indicate an increased sexual assault risk in comparison to sober women (Davis et al. 2009), non-drinking females may be more aware of dangerous or unpleasant situations such as sexual harassment. As a result of these differences regarding the perception of non-drinkers based on gender, unique consequences, and the identification of a clear knowledge gap, this study will focus specifically on the experiences of female non-drinkers, to provide an in-depth exploration of a clearly defined under-researched group of non-drinking students.

This study uses interpretative phenomenological analysis (IPA; Smith and Osborn 2003) to explore the lived experiences of non-drinking, female, first year undergraduates in the UK. Using IPA methodology allows for an in-depth analysis of how each participant makes sense of their own unique experiences. Given the dangers of binge drinking amongst UK undergraduates, it is imperative to explore the experiences of non-drinkers, unveiling their hardships and understanding the potential challenges this subgroup face when trying to make appropriate alcohol choices, particularly when their decisions subvert the cultural norm. This has wider relevance to all undergraduates who intend to reduce alcohol intake to begin to understand the challenges they may face if they too decide to abstain from alcohol. This research will build upon the small body of qualitative research on the topic of non-drinking students, generating further knowledge.

## Method

### Design

Eight, female, non-drinking first year undergraduates took part in a semi-structured interview, in which open-ended questions were used encouraging detailed experiential accounts. The data were transcribed verbatim, and subsequently analysed using interpretative phenomenological analysis (IPA; Smith and Osborn 2003).

### Participants

Participants were recruited through purposive sampling, obtaining participants by advertising via an online participant pool at the University of Lincoln. Inclusion criteria stipulated that individuals were female, an undergraduate and a non-drinker (defined as someone who either has never drank alcohol, or has only consumed alcohol once in the previous year).

Eight female, non-drinking undergraduates participated. The mean age of the participants was 21.5 years (SD = 5.29 range = 18–33 years). All participants had not drank alcohol for approximately 2 years or more. To protect anonymity, names of all participants have been replaced with pseudonyms (Table 1).

**Table 1:** Participants

Pseudonym	Age	Reason(s) for Non-Drinking Lifestyle
Laura	18	Witnessed negative effects of alcohol on family members.
Hannah	19	Religious constraints.
Elizabeth	33	Lost interest in drinking at age 18; dislikes nightclub environment.
Rachel	20	Lives healthy lifestyle; dislikes taste.
Bethany	25	Sickness phobia; alcohol not necessary to have a good time.
Jessica	18	Medication; negatively affected mood.
Dannielle	19	Social anxiety; not wanting to lose control of actions.
Megan	18	Did not enjoy taste; not wanting to lose control of actions.

### Data Collection

Ethical approval was obtained from the School of Psychology Research Ethics Committee (SOPREC) at the University of Lincoln (Ethical Clearance Number: 112015). Informed consent was obtained from all participants included in the study prior to data collection. Data were collected through semi-structured interviews, conducted within a comfortable and private room on the university campus.

In total, 15 open-ended interview questions were asked (e.g., “Tell me about your experiences as a non-drinking student”). Given the sensitive nature of the study, questions were tailored to be sensitive and respectful, whilst being clear and concise for the interviewee to easily understand. An interview schedule acted as a guide to structure the interview, to enable participants to direct the interview focus where possible. Interviews lasted on average 41 min. At the end of the interview, all participants were debriefed, thanked for their participation, and given the opportunity to ask questions.

### Data Analysis

Data were analysed in accordance with the principles of interpretative phenomenological analysis (IPA: Smith and Osborn 2003). Small sample sizes are consistent with IPA’s aim, as its idiographic nature enables exploration of each case individually, to generate rich and detailed descriptions of each participant’s unique experience first, before identifying commonalities across all cases. IPA emphasises not only that the research process involves participants trying to make sense of their own experiences, but also that the researcher is trying to make sense of the participant trying to make sense of their own experiences (Smith 2015). IPA also acknowledges that the process of interpretation relies on the participant’s capacity to communicate their experiences sufficiently, plus the researcher’s reflective capabilities to decipher what is being said.

The analytic process consisted of several stages. First, each interview was transcribed verbatim. Second, transcripts were read multiple times to gain familiarity and a thorough understanding of the content. Second, preliminary notes concerned key features of experience and participants use of language were recorded on each transcript. Third, data was explored systematically to identify emergent themes. Three superordinate themes from this analysis are presented in this article.

## Findings

Analysis revealed three super-ordinate themes: “Managing the feeling that you don’t belong”, “experiencing social exclusion” and “experiencing peer pressure and social stigma”. These themes represent the essence of the experiences of the participants in this study.

### “Managing the feeling that you don’t belong”

After asking participants about their experiences when attending social events, they explained the types of coping strategies they implement when faced with social pressures. Strategies include providing illegitimate excuses for not drinking or withdrawing themselves from social interactions with peers.

Many participants commented that being a non-drinker in an environment where drinking alcohol is engrained within its culture leads to difficulties when attempting to socialise with peers. All eight participants explained that not drinking in university was a lifestyle choice deemed abnormal or “some sort of disease” (*Megan*)*.*

Many expressed feelings of social isolation, feeling as though their peers do not want to socialise with them. Participants intentionally deceived their peers to gain acceptance. In social situations, participants lied about their drinking status, misleading their peers to believe that abstinence was only temporary, to alleviate the risk of peer scrutiny. In the following extract, Bethany employs deception. She uses an excuse as a method of escaping from a potentially negative social encounter:

- Extract 1—*If I know people are going out and they want me to go, I have been known to get the crutches out and use them and be like nah, I’m in too much pain (…) I’ve actually been known to explain to people, (…) that I have medical reasons and that’s why I can’t drink because it’s just easier than trying to explain to somebody that you don’t want to drink. (…) The second you mention medical they go, oh right you know and they back off (…) they’re not so keen to pressurise you (…) it’s just easier than trying to say look I don’t wanna drink and I’m not gonna drink.* (Bethany, 25)

Bethany’s social experiences appear to be particularly strained, as she describes her attempt to alleviate scrutiny and peer pressure. She finds it easier to pretend that she cannot drink for medical reasons, as it is easier than explaining that “you don’t want to drink”. This infers that past conversations about her non-drinking lifestyle have received unfavourable reactions. Peers may not have been accepting of her being a non-drinker, leading Bethany to feel she has to adopt the identity of being disabled with pain. She goes to the length of feigning an injury to provide a socially acceptable justification for her non-drinking. Using crutches as a prop to increase the legitimacy of her excuse, Bethany prevents being pressurised to drink alcohol. She explains that providing a medical reason for not drinking, instead of saying that she simply does not want to, reduces the risk of confrontation from others and avoids revealing her true non-drinking identity.

Many participants expressed disapproval for parties and nightclubs, because alcohol consumption was the dominating feature of the event. Many felt uncomfortable in these environments, which led to attempting to avoid attending future events of this type. Participants explained that the beginning of the academic term is seen as an opportunity to make new friends and socialise with new housemates; therefore, offers to attend alcohol-related events were frequent. They expressed that trying to decline invitations from their peers and new housemates was challenging, as this needed to be performed strategically without causing offence. Many felt that it was difficult to decline invitations as drinking students were described as having “strong personalities” (*Danielle*) and could be very persuasive. Excuses and tentative replies to offers of alcohol-related activities have been used as a means of managing these challenges. Jessica drew attention to the avoidance strategy she used:

- Extract 2—*That’s how people are social. My flatmates would ask me (…) are you coming out tonight (…) when I say “no, I’ll give this one a miss”, (…) it makes me feel really antisocial. Every time I say no, it gives off the message that I don’t wanna be social and they’ll stop asking me. If they ask me in the morning (…) it’ll be “I’ll think about it”, then in the evening I’ll be like “I’ll have an early night”. I find it quite difficult ‘cause it’s me saying I don’t want to do this with you is being personal.* (Jessica, 18)

In claiming that refusing an invitation will “give off the message that I don’t wanna be social and they’ll stop asking me”, Jessica implies that she feels challenged by her inconsistent mind-set. She wants to avoid joining in with pre-drinking or attending nightclubs with her housemates, yet, she still wishes to be asked, due to the risk of social exclusion. Jessica highlights how she believes that the process of social bonding within the university culture takes place solely through drinking*.* This is a reasonable assumption for Jessica to make, given previous literature illustrates that first year students use alcohol to facilitate social interaction (Penny and Armstrong-Hallam 2010).

Through subtle refusal, Jessica feels as if her housemates will take her rejection personally, thus souring her relationship with them. Her tentative replies and reluctance to say no suggest that she uses this technique to hide her disinterest in drinking alcohol. Her avoidance strategies extend to withdrawing herself from further interaction with her housemates through isolating herself in her bedroom. This prevents insistence to go drinking and avoids conflict that may arise as a result of her refusal. Jessica’s use of an avoidance strategy enables her to manage the challenges in social situations; if she can avoid conflict then this will ensure she will not be excluded permanently from her peer group.

### “Experiencing Social Exclusion”

In addition to difficulties in social management, all participants in this research highlight the impact that non-drinking has on their ability to form friendships and socialise comfortably with others. All participants explain difficulty in finding opportunities to socialise with their peers at university. As previously explained, Fresher’s Week is one of the main methods of meeting new people at the start of the academic year; participants in this research confirm this, highlighting its social significance. However, their experiences of Fresher’s Week were significantly restricted. Being a non-drinker resulted in exclusion from social opportunities, especially during the start of their university experience. When the researcher asked Megan about the activities available for non-drinking students in the local area, she acknowledged that opportunities to socialise without alcohol were scarce:

- Extract 3—*Before I came to uni (…) I asked a second year (…) I said oh is there anything for non-drinkers but (…) they couldn’t really think of any or couldn’t remember any. I know they could do more. The only thing that wasn’t clubbing or entirely alcohol related was the quiz, which I went to (…) But that was the only thing and I mean one night out of a whole week which is built up and it’s Fresher’s Week, it’s meant to be a big thing, so I feel like I missed the boat a little bit there.* (Megan, 18)

Megan explained that she attended only one event during Fresher’s Week, as only one activity was not alcohol based. This was the view of all eight participants in this research; they all reported that the majority of the activities in the city revolve around heavy drinking.

Megan sought to find events that she could attend; however, current students were unaware of any activities that she would be able to go to. This may be due to a lack of activities available or a lack of awareness from other students, as it is reasonable to assume that the university may not advertise activities that will not attract the majority of students. For those who are abstainers or light drinkers, the scarcity of non-drinking activities may act as a barrier to meeting new people. Megan also indicates feels she was unable to take advantage of the social opportunities that Fresher’s Week provided.

In line with all other participants interviewed, Megan believes the university should cater for the needs of first year, non-drinking students by hosting and increasing the promotion of alternatives to alcohol-dominated events. These events need to be promoted during Fresher’s Week to ensure that the week is equally inclusive for both drinkers and non-drinkers and provides all first year students ample opportunity to socialise and bond with new people.

Being a non-drinking student has a considerable impact on the ability to make and maintain friendships. For all participants interviewed, making friends was a significant challenge. One student explained that she could count her number of friends “on one hand” (*Bethany*). Many expressed that the behaviour and personality of their peers changed when intoxicated. This meant that when trying to form a bond, participants felt that the friendship was not genuine, because the drunken individual would not be able to remember former interactions or conversations that occurred between them. Danielle expressed the importance of feeling part of a friendship group, but has struggled with this as a result of being a non-drinker:

- Extract 4—*I’ve definitely struggled to make friends and I still feel like I haven’t made a proper group of friends and I see everyone else with lots of friends (…) I don’t think alcohol is the blame for that but I think it would have been easier if I had been like that (…) I definitely think it’s harder to fit in. I feel everyone formed friendships now and it’s like too late to start going in to them, so I feel like I just don’t fit in with them*. (Danielle, 19)

In this extract, Danielle expressed that she feels she does not belong to a social group; she observes others making friends, but this is primarily due to alcohol enhancing their ability to do this. She does not blame alcohol for her inability to form friendships, but explains that if she were a drinker, then this would have been an easier task. It is implied that being a non-drinker makes it particularly difficult to establish a social network.

This interview was conducted 3 months after the participants started university. Danielle implies that to join or establish a friendship group, she would have to do so quickly otherwise she will have missed her opportunity. Danielle emphasises the pressure to make friends, implying that if she cannot make friends within her first term, then she may not be able to for the remainder of her time at university. This highlights an additional concern expressed by Danielle and other interviewees; the risk of isolation. Another student expressed that she felt lonely and “isolated at times” (*Bethany*)*.* Danielle felt as though she does not “fit in with them”, acknowledging “them” to be a distinct social group of which she was not a part. This demonstrates a divide between drinking students and non-drinking students and the differentiation between their behaviours and personalities.

### “Experiencing peer pressure and social stigma”

All participants expressed their views on their binge drinking peers, focusing on their experiences of the way in which peers who drink alcohol would comment on their personality as a result of being a non-drinker and the way they would make them feel.

All eight participants interviewed expressed that interactions with drinking students were often unpleasant. As a result of university drinking culture, it was highlighted that, as a first year undergraduate, students were “expected to go out and get drunk” (*Megan*) and conform to the typical student lifestyle. This deviation from the norm, led to the requirement of justification and explanation as to why they would not be drinking. One participant hypothesised that this was due to the fact that “not everybody understands the concept of not drinking” (*Rachel*). Jessica describes the extent to which, on one occasion, peer pressure led her to drinking alcohol.

- Extract 5—*They’d be like, you really do want to though don’t you, they’d pour a drink out for me, sort of say oh just have a drink, (…) well the second time I went out I sort of caved into that.* (Jessica, 18)

Jessica highlights her inability to refute peer pressure, as she allowed herself to drink alcohol, despite being originally opposed to doing so. The process of pouring out drinks, an active offer of alcohol, is evidence of direct encouragement, the most direct form of peer pressure (Santor et al. 2000). It is implied that by conforming, Jessica alleviated potential teasing and established peer acceptance and feelings of belongingness with her new housemates. This extract also highlights Jessica’s lack of confidence and capability to resist social pressure, as she presents lowered self-regulatory self-efficacy in order to manage social pressures, hence why she cooperated.

In contrast, other participants expressed experience of social aggression in a different manner. All participants felt that they were perceived as unsociable or boring, with seven out of eight students subject to hurtful comments either to their face, or behind their backs. During a university society meeting, one participant, Laura, was asked whether she drank alcohol. When she said no, a member of the society meeting spoke about Laura negatively to another group member behind her back. At a later date, the girl who made the initial negative comments about Laura said she mistook her for a boring person, just because she was a non-drinker. Laura gives an account of her experience:

- Extract 6—*They asked us in a group, “Do you drink? Do you wanna go out?” I was like I don’t drink, so I don’t go out (…) Then I think she basically told someone else (…) “she’s quite boring isn’t she cause she doesn’t drink” and “who doesn’t drink at uni, drinking’s like the whole life of uni” (…) The thing is the girl who said I’m quite boring (…) she opened up (…) she was just like I didn’t really expect you to be like this (…) I kind of like thought you’re just someone who doesn’t drink so I just thought you didn’t really go out much so I just thought you were quite a boring person.* (Laura, 18)

Unlike other participants in this study, Laura felt confident admitting to being a non-drinker within a group. However, this act of confidence led to social aggression from her peers, and she was immediately subject to scrutiny, negative comments, and labelling. Drinkers made an immediate interpretation of Laura’s personality, linking non-drinking with being boring and unsociable. It is implied that Laura is in fact an outgoing, social person, and her personality defies the lingering non-drinker stereotype. It seems that alcohol played a central role in influencing alcohol-consuming peers to make an immediate judgement of Laura, instead of coming to a more rounded, reasonable understanding of Laura’s real self; her true personality and sociability are misunderstood and hidden as a consequence.

Laura also implies that her peers may be mocking her lifestyle choice. The drinking student classes alcohol as “the whole life” of university which suggests that they view Laura’s non-conformity as atypical. Laura explains that the individual who was judgemental towards her had an expectation about Laura’s personality, based purely on her teetotalism and deviation from a social norm. This extract highlights the judgement non-drinkers face whilst attending university, experiences that only occur as a result of non-conformity to a typical student lifestyle. It is this deviation from the typical university lifestyle and cultural norm that caused students in this study to feel uneasy starting university. Megan sheds lights on her feelings:

- Extract 7—*I was really worried about coming to uni because I don’t drink. I didn’t want to get to uni and them to think I was boring for not drinking. I was really nervous about it*. (Megan, 18)

It is implied here that before starting at university, Megan was aware of the predominating alcohol norms and expectations when attending university in the UK, which as a non-drinking individual caused her to experience apprehension about starting university. She was concerned about the existing social stigma of being a non-drinker and did not want to be classified or stereotyped as boring by her university peers. This may potentially be due to the fact that these existing perceptions of non-drinker characteristics could leave her automatically susceptible to feelings of alienation and social acceptance during the start of her university experience. It is possible that her peers may treat her differently for being a non-drinker, which caused Megan angst, and she may not be given adequate opportunity to behave in a way that represents her true self when meeting new people as a consequence of existing stereotypes.

## Discussion

This study provides a valuable contribution to the understanding of the lived experiences of female, non-drinking students during their first year of undergraduate study attending a UK university. Through the use of IPA methodology, the research provides a rich understanding of the challenges experienced by this sample, adding to the small body of literature on the topic.

It was evident from all interviews that non-drinkers acknowledge that alcohol consumption is a dominant norm amongst university students in the UK, which plays a significant role in social environments. The decision not to drink alcohol by these young women has led to them learning how to prepare for and respond to social pressures, people’s expectations, and the consequences of being a non-drinker in an excessive drinking culture. Many were unsure how to handle non-conformity to cultural norms, leading to the development of coping strategies to manage peer pressure to drink alcohol or to engage in the prevailing alcohol fuelled student life such as pre-drinking. This study highlights that despite being a female non-drinker, which despite literature indicating that in some situations is deemed as more tolerated, they too experienced considerable challenges with peer acceptance.

Given that alcohol is a key tenet for social interaction within the undergraduate population (Gill 2002), being a non-drinking first year student meant that meeting and interacting with drinking peers caused apprehension. Many were concerned about how they would be perceived by others and the potential consequences this would cause when making new friends as a result of the existing expectations and assumptions of non-drinkers personalities and behaviours. In addition, participants felt as though their lifestyle choice was questioned and during conversations with peers about their non-drinking, they felt judged for choosing not to drink, coming to the assumption that drinking students do not understand the concept of non-drinking. As Social Identity Theory (Taijfel and Turner 1986) suggests, this may be a result of drinking students making distinctions between themselves, as the in-group, and non-drinkers as the outgroup. These results chime with Conroy and de Visser (2015), in which their participants expressed frustration having to justify their decision not to drink and having to reveal personal accounts of why there are a non-drinker during conversations with new people.

For this sample of female UK undergraduates, it seemed important to seek acceptance from their peers. As a result of identifying as and presenting themselves as a non-drinker caused concern for participants, and led to having to strategically manage social situations, as it was easier to mislead new acquaintances about their non-drinking status as an opposed to admitting to being a non-drinker. Through finding excuses or avoiding uncomfortable conversations about drinking, participants would not only avoid peer pressure and encouragement to drink, but would also reduce the risk of being excluded from social events with new acquaintances altogether. Our findings are similar to Conroy and de Visser (2015) in which participants revealed that passing as an alcohol consumer, such as by holding an alcoholic drink was an easier and sensible method as opposed to drawing attention to their non-drinking behaviour.

These findings also draw comparisons with previous research that highlighted difficulties for non-drinking students with regard to the social adversities and pressures that can occur when identifying as a non-drinking individual (Conroy and de Visser 2014; Herring et al. 2014; Piacentini and Banister 2009). Although there are limitations in comparing previous literature to this study, for example the differing samples used, it is helpful to show a consistency of non-drinkers’ challenging experiences across a university population.

### Implications and Future Research

The analytical findings reveal that the absence of alcohol hinders the bonding process for female, first year undergraduate students, resulting in an unfavourable social experience at the start of the university journey. These challenges occurred as a result of limited opportunities to socialise when alcohol was not the dominating feature of the event. All participants were critical of the activities provided by the university, both during Fresher’s Week and subsequent weeks. Students highlighted events hosted were unenjoyable and unsuitable for non-drinkers to attend, resulting in this group of students missing out on socialising, causing feelings of loneliness. It is vital that this is addressed by the university, ensuring that a larger number of non-alcohol events are hosted and promoted to help towards alleviating the documented challenges for non-drinkers, ensuring that the needs of this small minority of students are not overlooked. For example, it would be important to ensure that there was a broader range of Fresher’s Week activities, through offering an alternative alcohol-free event for each night of Fresher’s Week. Furthermore, for those who make the decision to reduce their alcohol intake, the wider range of activities available to attend on campus will potentially make their decision to reduce their alcohol intake easier, and motivate others to consider changing their drinking behaviours if they know that in doing so, their social life will not be as constrained.

The results of this study highlight the importance of focussing on inclusivity, working towards an attitude shift where drinking students respect non-drinkers’ decision to abstain from alcohol. It can be hypothesised that the promotion of alternatives to alcohol fuelled events may reduce segregation between drinkers and non-drinkers, enabling them to socialise together comfortably. In encouraging drinkers and non-drinkers to socialise more frequently together, this may potentially challenge peers’ existing negative constructs of non-drinkers and their behaviours. A small proportion of universities in the UK have begun to acknowledge the needs of non-drinking students, through introducing alcohol-free zones in London-based universities (BBC News 2012); however, no real attempt has been made to work towards increasing inclusivity of non-drinkers nationwide. Future research might address this exact issue to determine how universities can meet the social needs of non-drinkers both on campus and within the local community. Potential changes should aim to shift the focus from alcohol-fuelled events to alternative night time events, such as mocktail events, quiz nights or night tours of the city. Focus group data can help universities to understand the changes required and act upon the recommendations made by the students themselves, in order to make students feel more comfortable on campus and in the local community.

Based on the methodology of IPA, the use of a small, homogenous sample has positively provided clarity and an in-depth exploration regarding the lived experiences of female non-drinking students in their first year of undergraduate study; however, this homogenous sample also questions the study’s generalisability. This means that other groups of non-drinking students may understand their experiences as a non-drinker and manage being a non-drinker in social situations differently or more successfully. Despite this, through the idiographic nature of IPA, we have illuminated the importance of continuing to research into the phenomenon of non-drinking behaviours at university and the avenues in which to explore. For example, it would be particularly interesting to explore the experiences of female non-drinkers over the 3 years at university. Through qualitative methods, future longitudinal research could aim to examine the differences between the experiences of students in their first year, to those in their third year of study. The objective would be to collect data through focus groups to understand if the experiences are different (i.e., better or worse), across year groups, and in what way being a non-drinker has impacted them across the years. In addition to continuing qualitative research in this area, quantitative measures can aim to build on qualitative findings to develop a wider picture of the experiences of female non-drinking undergraduates on a larger scale. If the consensus is that a large proportion of students are experiencing social difficulties whilst at university, these findings can help UK universities to ensure that appropriate support is in place for students who are struggling to cope with the stressors of university, including being equipped with the skills to successfully manage challenging situations, including handling peer pressure.

## Conclusion

Building on a small body of literature, this study has presented an account of the lived experiences of eight non-drinking, female UK students, in their first year of undergraduate study. The use of interpretative phenomenological analysis allowed for an in-depth, idiographic exploration of this particular group of students. Findings have highlighted that subversion of the cultural alcohol norms within a university setting can result in female non-drinkers living and learning to live with challenges including social constraints, peer pressure, and criticisms from peers regarding leading a non-drinking lifestyle. Whilst these findings related to a small group of non-drinking students, this study has highlighted the importance of focusing research attention towards non-drinking students. Beneficially, this research provides an understanding of the potential challenges that may occur if an undergraduate decides to abstain from alcohol, which can help university to reconsider not only the support that should be available to those who are struggling to manage with their university journey, but also provide a range of non-alcohol dominated events to ensure all students feel socially inclusive.
